# Caspr2 interacts with type 1 inositol 1,4,5-trisphosphate receptor in the developing cerebellum and regulates Purkinje cell morphology

**DOI:** 10.1074/jbc.RA120.012655

**Published:** 2020-07-16

**Authors:** Liam Argent, Friederike Winter, Imogen Prickett, Maria Carrasquero-Ordaz, Abby L. Olsen, Holger Kramer, Eric Lancaster, Esther B. E. Becker

**Affiliations:** 1 Department of Physiology, Anatomy and Genetics, University of Oxford, Oxford, United Kingdom; 2 Department of Biochemistry, University of Oxford, Oxford, United Kingdom; 3 Department of Neurology, University of Pennsylvania, Philadelphia, Pennsylvania, USA

**Keywords:** calcium, cerebellum, inositol 1,4,5-trisphosphate (IP_3_), inositol trisphosphate receptor (InsP_3_R), dendrite, development, synapse, Caspr2, Cntnap2, Purkinje cell

## Abstract

Contactin-associated protein-like 2 (Caspr2) is a neurexin-like protein that has been associated with numerous neurological conditions. However, the specific functional roles that Caspr2 plays in the central nervous system and their underlying mechanisms remain incompletely understood. Here, we report on a functional role for Caspr2 in the developing cerebellum. Using a combination of confocal microscopy, biochemical analyses, and behavioral testing, we show that loss of Caspr2 in the *Cntnap2*^−/−^ knockout mouse results in impaired Purkinje cell dendritic development, altered intracellular signaling, and motor coordination deficits. We also find that Caspr2 is highly enriched at synaptic specializations in the cerebellum. Using a proteomics approach, we identify type 1 inositol 1,4,5-trisphosphate receptor (IP_3_R1) as a specific synaptic interaction partner of the Caspr2 extracellular domain in the molecular layer of the developing cerebellum. The interaction of the Caspr2 extracellular domain with IP_3_R1 inhibits IP_3_R1-mediated changes in cellular morphology. Together, our work defines a mechanism by which Caspr2 controls the development and function of the cerebellum and advances our understanding of how Caspr2 dysfunction might lead to specific brain disorders.

Contactin-associated protein-like 2 (Caspr2) is a member of the contactin-associated protein family that belongs to the neurexin superfamily of proteins (1). Similar to classical α neurexins, Caspr2 has a short C-terminal intracellular domain, a single transmembrane helix, and a large N-terminal extracellular domain (ECD). However, Caspr2 contains multiple ECD domains not found in classical neurexins and is thought to adopt a different three-dimensional structure (2, 3), likely mediating distinct functional protein-protein interactions. Caspr2 function was first assessed in the peripheral nervous system (PNS), where it was found to form a cell adhesion complex with Contactin-2 and cluster voltage–gated potassium channels at juxtaparanodes (1, 4). Since then, mutations in the *CNTNAP2* gene, encoding Caspr2, have been associated with a range of neurodevelopmental cognitive disorders including autism spectrum disorder (ASD), dyslexia and language impairment, epilepsy, and schizophrenia (5–8). However, surprisingly little is known about the role of Caspr2 and its potential interaction partners in the central nervous system. Studies to date have mostly focused on the consequences of disrupting Caspr2 function on the balance between excitatory and inhibitory circuits and synapse formation in the cerebral cortex (9–11). The role of Caspr2 in other brain areas has remained unexplored. Notably, polymorphisms in *CNTNAP2* have been associated with reduced cerebellar gray matter in humans (12). Similarly, imaging studies in *Cntnap2* knockout mice have reported an association between the homozygous deletion of *Cntnap2* and altered cerebellar size (13). Moreover, autoantibodies against Caspr2 have been linked to cerebellar ataxia in some patients with anti-Caspr2 antibody–associated encephalitis (14–16). Thus, evidence from both patients and mice points toward a possible function for Caspr2 in the cerebellum.

In this study, we find that Caspr2 is enriched at synapses in the developing cerebellum. Mutant mice lacking *Cntnap2* show distinct cerebellum-associated behavioral impairments and abnormal Purkinje cell development. Interestingly, we identify type 1 inositol 1,4,5-trisphosphate receptor **(**IP_3_R1) as a specific synaptic interaction partner of the Caspr2 ECD in the molecular layer (ML) of the developing cerebellum. The interaction of the Caspr2 ECD with IP_3_R1 inhibits IP_3_R1-mediated changes in cellular morphology. Together, our findings suggest that Caspr2 regulates the development of Purkinje cell dendrites by interacting with IP_3_R1 at synapses, suggesting a novel mechanism by which Caspr2 dysfunction might lead to aberrant cerebellar behaviors and associated neurological disorders.

## Results

### Caspr2 is expressed at the synapse in the developing cerebellum

**Figure 1.**
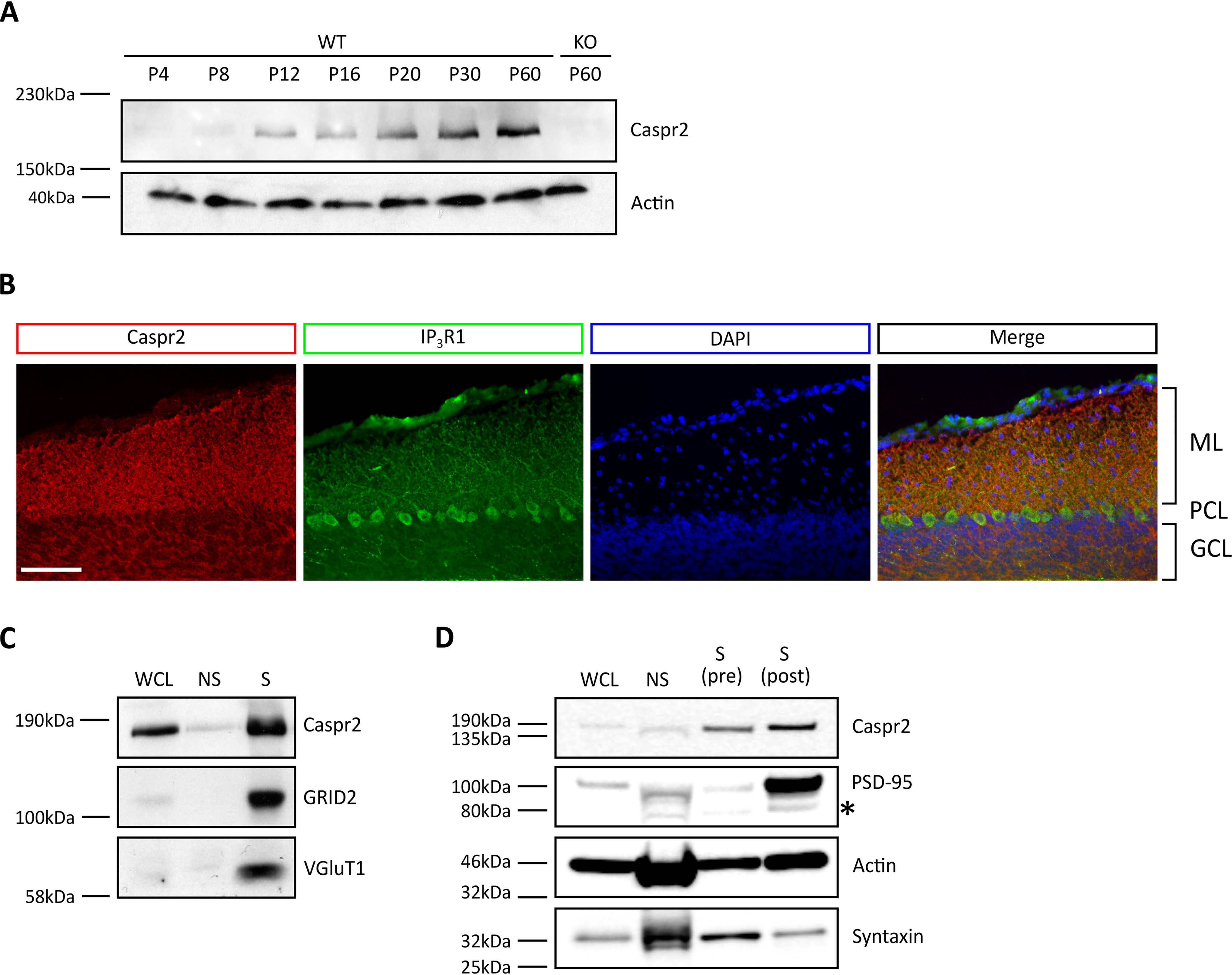
**Caspr2 is expressed in the developing and adult cerebellum.** *A*, temporal expression pattern of Caspr2 in the murine cerebellum, during postnatal development and adulthood. Protein was extracted from Caspr2 WT and KO cerebellum at the indicated time points and analyzed by immunoblotting for Caspr2 and the loading control actin. *B*, Caspr2 is enriched in the ML. Shown are representative images of immunostained P20 WT mid-sagittal cerebellar sections. Individual sections were triple-stained with antibodies against Caspr2 (*red*) and IP_3_R1 (*green*) and the DNA dye DAPI (*blue*). *Scale bar*, 100 μm. *C*, Caspr2 is enriched at synapses in the developing cerebellum. Synaptosomes were isolated from P14 WT cerebellum, lysed, and then subjected to immunoblotting for Caspr2 and the synaptic markers GRID2 and VGluT1. *D*, Caspr2 is present at both pre- and postsynaptic specializations. Western blot analysis of P14 WT cerebellum subjected to subsynaptic fractionation followed by immunoblotting for Caspr2; syntaxin, a marker of presynaptic specializations; PSD-95, a marker for postsynaptic specializations; and actin as loading control. All images shown are representative of at least three independent experiments (biological replicates). Nonspecific immunoreactivity is indicated by an *asterisk*. *KO*, *Cntnap* knockout; *P*, postnatal day; *ML*, molecular layer; *PCL*, Purkinje cell layer; *GCL*, granule cell layer; *WCL*, whole-cell lysate; *NS*, nonsynaptic; *S*, synaptic; *S (pre)*, presynaptic specialization; *S (post)*, postsynaptic specialization.

We first investigated the temporal and spatial expression pattern of Caspr2 in the developing cerebellum. Caspr2 protein was highly expressed in the mouse cerebellum from the second week of postnatal development (Fig. 1*A*), which coincides temporally with the outgrowth of Purkinje cell dendrites and synapse development in the cerebellar cortex (17). Caspr2 protein expression remained high in the adult cerebellum (Fig. 1*A*), consistent with earlier studies (18). We then assessed where in the mouse cerebellar cortex Caspr2 was expressed, focusing on the developing cerebellum. Mid-sagittal sections of postnatal day 20 (P20) mouse cerebellum were immunostained with antibodies against Caspr2 and IP_3_R1. The latter is specifically expressed in Purkinje cells (19) and was used as a marker to define Purkinje cells, alongside their characteristic morphology. The strongest Caspr2 signal was detected in the ML, where developing Purkinje cells make synaptic contacts with granule cell parallel fibers and in the granule cell layer (Fig. 1*B*). This is consistent with the presence of Caspr2 in the ML of the adult cerebellum (18). To confirm the localization of Caspr2 to synapses, we carried out fractionation experiments. Cerebellar synaptosomes were prepared from 2-week-old mouse cerebellum, lysed, and analyzed by Western blotting. Caspr2 was found to be highly enriched in the synaptic fraction (Fig. 1*C*). To identify the cerebellar subsynaptic compartments in which Caspr2 is present, we isolated pre- and postsynaptic specializations from P14 mouse cerebellum. Surprisingly, Caspr2 was found in both pre- and postsynaptic compartments (Fig. 1*D*). Together, these results suggest that Caspr2 is highly expressed in the developing and adult cerebellum and enriched in multiple synaptic compartments.

### Caspr2 has a functional role in the cerebellum

**Figure 2.**
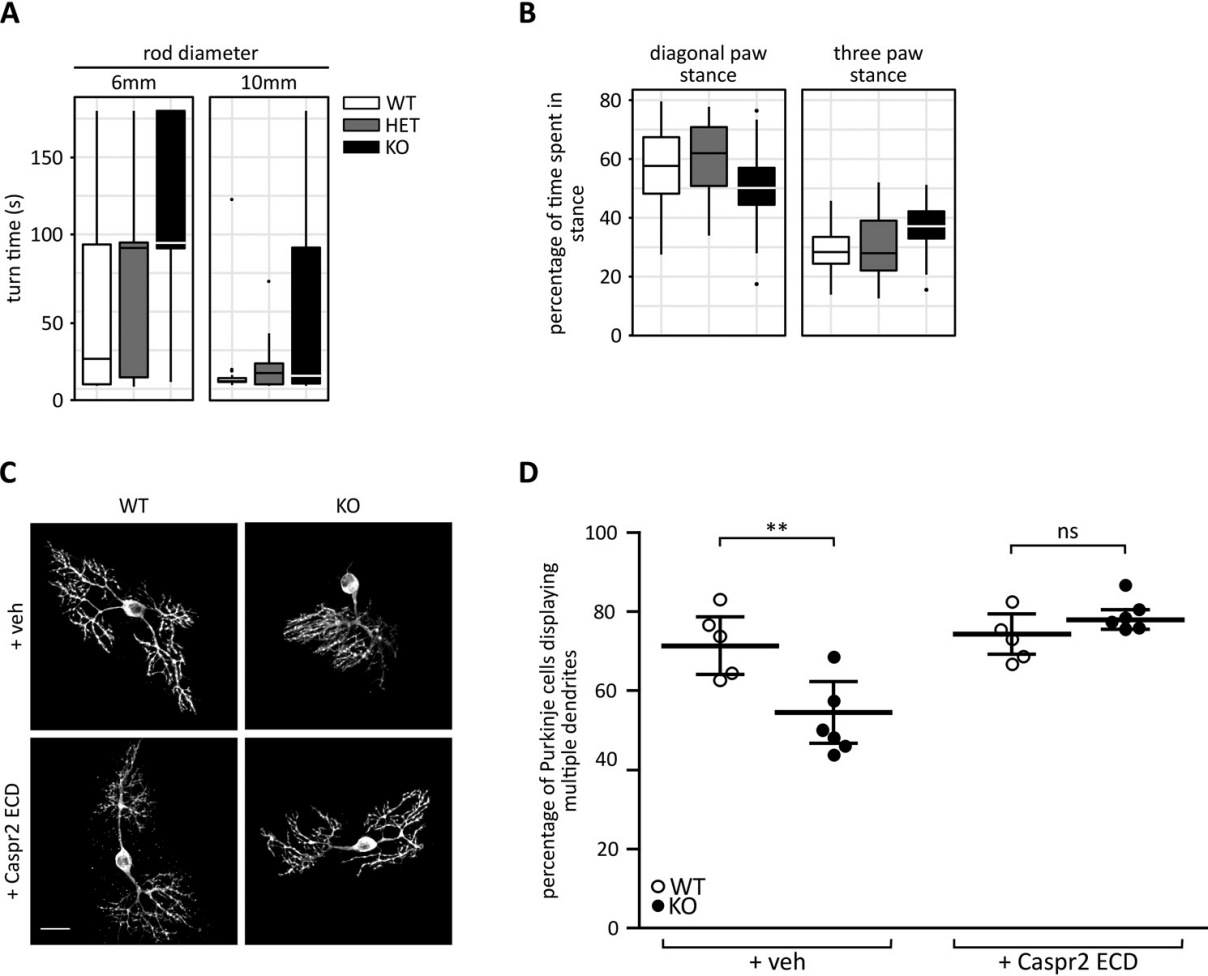
**A functional role for Caspr2 in the cerebellum.** *A*, KO mice take longer to turn around while navigating a static rod than WT and HET littermates. *p* < 0.05, Kruskal–Wallis χ^2^ test. *n* = 8 female and 9 male (WT), 10 female and 10 male (HET), and 10 female and 10 male (KO). *B*, KO mice spend more time supporting themselves on three paws than WT and HET littermates. *p* < 0.0001 for time spent in the diagonal paw stance; *p* < 0.00001 for time spent in the three paw stance, effect of genotype in a three-way (age, sex, genotype) mixed-model analysis of variance. *Box and whisker plots* for *A* and *B* show median, interquartile range, and interquartile range × 1.5. Outliers are indicated by *black circles*. *n* numbers are as described above; however, data for five animals (1 female WT, 1 female and 2 male HET, and 1 female KO) were excluded from one data set (3 months) due to an accidental change in recording parameters. *C*, organotypic cerebellar slice cultures from P9 WT and KO littermates were cultured for a week and dosed with either recombinant ECD Caspr2 or vehicle (10 mm Tris, pH 8, 200 mm NaCl) before fixation and immunostaining for IP_3_R1. *Scale bar*, 25 μm. *D*, significantly fewer Purkinje cells from KO animals display multiple dendrites compared with WT littermates. This effect is rescued by the addition of recombinant Caspr2 ECD. *n* = 5 (WT) and 6 (KO). Data are presented as a scatterplot with mean ± S.D. superimposed. **, *p* < 0.01; *ns*, not significant, regular two-way analysis of variance with Sidak's multiple-comparison post hoc test. *veh*, vehicle; *KO*, *Cntnap2* knockout.

The finding that Caspr2 is strongly expressed in the cerebellum, combined with the description of phenotypes indicative of cerebellar deficits including motor deficits and language impairment in patients with reduced Caspr2 function (7, 8, 15), led us to investigate whether mice deficient in Caspr2 would display any distinct cerebellar deficits. Motor coordination and gait was tested in *Cntnap2* mutant animals and WT littermates using the static rod test and the CatWalk system, respectively. For the static rod test, animals were placed at the end of the rod, facing away from the platform, and the time taken for them to turn around and face the platform was recorded. We observed a significant effect of genotype, with *Cntnap2*^−/−^ knockout (KO) mice taking longer to turn around than WT and *Cntnap2*^+/−^ heterozygous (HET) littermates (Fig. 2*A*). Analyzing the number of paws supporting the animals as they walk, we found a significant decrease in the proportion of time KO mice were supported by the diagonal pair of paws and a corresponding increase in the proportion of time the mice were supported by three paws relative to WT and HET littermates, indicating that the KO animals have an unstable gait (Fig. 2*B*). This effect of genotype was detected in both juvenile (P35–42) and adult mice. Taken together, these behavioral data suggest that Caspr2 is likely important for normal cerebellar function.

We next investigated Caspr2's function in the cerebellum at the cellular level. Given that Caspr2 protein expression starts within the second postnatal week, at which time the rapid growth and maturation of Purkinje cell dendrites occurs (17), we set out to investigate whether Caspr2 might play a role in the dendritic development of Purkinje cells. Organotypic cerebellar slice cultures were prepared from P9 mice and cultured for 7 days before being subjected to immunostaining using an antibody against the Purkinje cell marker IP_3_R1. Immature Purkinje cells often display multiple primary dendrites and perisomatic protrusions before they develop their ultimate dendritic tree (20). Interestingly, fewer Purkinje cells taken from KO mice displayed multiple primary dendrites than those taken from WT littermates (Fig. 2, *C* and *D*). This phenotype was rescued by the addition of recombinant Caspr2 ECD (Fig. 2, *C* and *D*). Together, these findings suggest a role for the ECD of Caspr2 in regulating early Purkinje cell dendritic development.

### Caspr2 interacts with IP_3_R1 at synapses in the developing cerebellum

**Figure 3.**
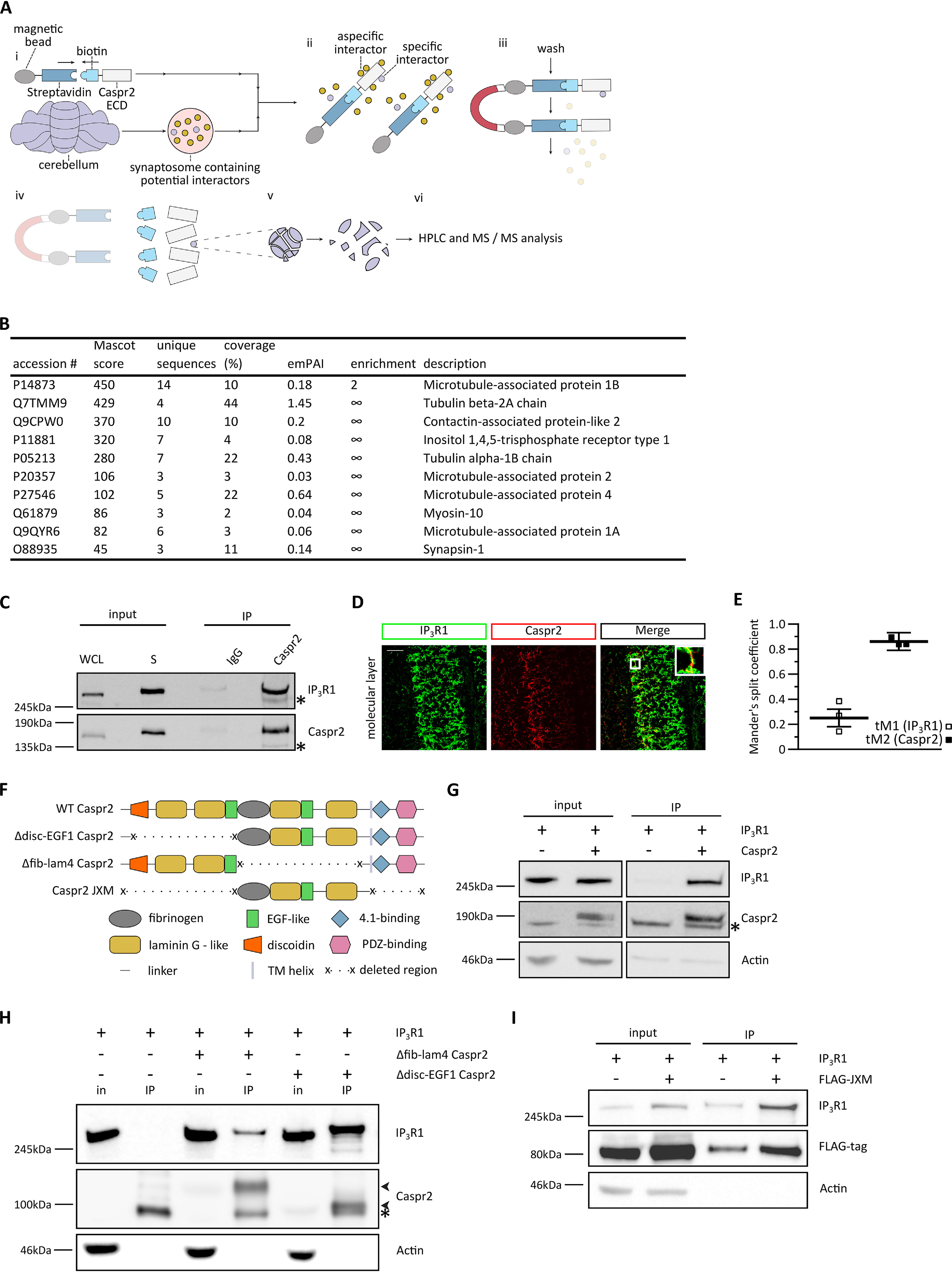
**Caspr2 interacts with IP_3_R1 at synapses in the cerebellum.** *A*, schematic illustration of the pulldown experiment protocol. *i*, assembly of bait protein and preparation of cerebellar synaptosomes; *ii*, synaptic proteins bind to the bait protein; *iii*, washing removes aspecific interactors; *iv*, elution; *v*, trypsinization of cognate interactors; *vi*, analysis of protein fragments. *B*, candidate Caspr2-binding partners identified by MS analysis of the pulldown experiment. Proteins were considered as interaction partners if 1) two or more unique sequences were identified and 2) the enrichment value was ≥2, where enrichment was calculated as emPAI(Caspr2 ECD-IP)/emPAI(mock-IP). *C*, synaptosomes were prepared from P14 WT mouse cerebellum and then subjected to immunoprecipitation with an anti-Caspr2 antibody or control IgG, followed by immunoblotting for Caspr2 and IP_3_R1. Images are representative of three biological replicates. *, nonspecific immunoreactivity. *D*, representative images of immunostained P14 cerebellar sections. Individual sections were double-stained with antibodies directed against Caspr2 (*red*) and IP_3_R1 (*green*). *Scale bar*, 50 μm; the *zoomed inset* is magnified ×4. *E*, average ML Mander's split coefficients ± S.D.: tM1 (IP_3_R1) = 0.250 ± 0.075; tM2 (Caspr2) = 0.856 ± 0.065. *n* = 3 (WT) animals, 4 unique images/animal. *F*, schematic illustration of the various Caspr2 constructs utilized. *G*, Caspr2 and IP_3_R1 co-immunoprecipitate from HEK293FT cell lysates. Lysates of HEK293FT cells transfected with IP_3_R1 alone or IP_3_R1 together with Caspr2 were subjected to co-immunoprecipitation with an anti-Caspr2 antibody followed by immunoblotting. *, nonspecific immunoreactivity. A representative image of three biological replicates is shown. *H*, lysates of HEK293FT cells transfected with IP_3_R1 and Caspr2 deletion constructs were subjected to co-immunoprecipitation with an anti-Caspr2 antibody followed by immunoblotting. Bands corresponding to Caspr2 deletion constructs are indicated by *arrowheads*. *, nonspecific immunoreactivity. A representative image of two biological replicates is shown. *I*, the Caspr2 JXM domain is sufficient for Caspr2-IP_3_R1 interactions. HEK293FT cells transfected with IP_3_R1 and FLAG-tagged Caspr2 JXM were subjected to co-immunoprecipitation with an anti- IP_3_R1 antibody followed by immunoblotting. A representative image of two biological replicates is shown. *In*, input; *IP*, immunoprecipitation; *S*, synaptic fraction; *WCL*, whole-cell lysate.

We next determined the molecular mechanism underlying Caspr2 function in the developing cerebellum. Whereas the Caspr2 ECD interacts with Contactin-2 at juxtaparanodes in the PNS (1) and other proteins have been reported to interact with the intracellular domain of Caspr2 (21, 22), no proteins have previously been reported to specifically interact with the ECD of Caspr2 at central nervous system synapses. To gain insight into the functional role of Caspr2 in cerebellar synapse development, we employed a proteomics approach. Recombinant, biotinylated Caspr2 ECD was coupled to streptavidin-coated beads and used to pull down interacting proteins from cerebellar synaptosomes prepared from P14 WT mice (Fig. 3*A*). To control for false positives due to nonspecific interactions, we used streptavidin-coated beads coated with contaminant proteins from the recombinant Caspr2 ECD purification. LC–MS/MS analysis of the pulldown experiment identified several candidate interaction partners (Fig. 3*B*; see Table S1 for full results). Interestingly, one of these interaction partners was the Ca^2+^ channel IP_3_R1, a key protein involved in cerebellar development and function. IP_3_R1 is located at the endoplasmic reticulum (ER) and plasma membrane in Purkinje cells (19, 23) and thus represents a plausible interaction partner for Caspr2. Nine separate peptide spectrum matches for IP_3_R1 were obtained in the test condition, compared with none in the control condition. Seven of the nine matches represented distinct sequences, with all seven being unique and significant (*p* < 0.05).

The interaction between Caspr2 and IP_3_R1 was validated in co-immunoprecipitation experiments. Both Caspr2 and IP_3_R1 were found to be enriched in cerebellar synaptosomes, and Caspr2 specifically co-immunoprecipitated with IP_3_R1 from cerebellar synaptosome lysates (Fig. 3*C*). To determine whether the spatial distribution of the two proteins was consistent with their possible interaction *in vivo*, cerebellar sections (P14) were immunostained using specific antibodies directed against IP_3_R1 and Caspr2 and imaged using confocal microscopy. Strong Caspr2 staining was observed in the white matter (WM) and in the deep ML, whereas IP_3_R1 was detected in the WM and the Purkinje cell layer and throughout the ML (Fig. 3*D* and Fig. S1*A*). The degree of co-localization was quantified in both the ML and the WM using Manders split coefficients (24) as this method permits the presence of additional, mutually exclusive binding partners, and IP_3_R1 is found in the ER and at the plasma membrane. Caspr2 and IP_3_R1 were found to co-occur in the deep ML, where the Mander's coefficient value for the Caspr2 channel was 0.856 (Fig. 3*E*). In contrast, much less co-occurrence was observed in the WM, where the Mander's coefficient value for the Caspr2 channel was 0.441 (Fig. S1*B*). A combination of the high density of axons in the white matter and the resolution of the imaging system used likely contributed significantly to this result. Taken together, these data are consistent with a specific interaction of Caspr2 and IP_3_R1 at synapses in the deep ML of the cerebellum.

We next carried out structure-function analyses to determine the regions of Caspr2 and IP_3_R1 that associate with each other. Caspr2 and IP_3_R1 were found to robustly interact in lysates from HEK239FT cells overexpressing Caspr2 and IP_3_R1 (Fig. 3*G*). Interestingly, the Caspr2-IP_3_R1 interaction was maintained when the co-immunoprecipitation was repeated in the presence of the Ca^2+^-chelating agent EGTA (Fig. S2), indicating that the interaction of the two proteins is not modulated by Ca^2+^ levels. IP_3_R1 interacted strongly with a mutated Caspr2 protein, in which the ECD domains distal to the membrane were deleted (Δdisc-EGF1), and only weakly with a Caspr2 mutant missing the membrane-proximal ECD domains (Δfib-lam4) (Fig. 3*H*). These results suggest that the four domains that comprise the membrane-proximal region of the Caspr2 ECD are necessary for the Caspr2-IP_3_R1 interaction. We termed this region the juxtamembrane (JXM) region. In further experiments, we found that the Caspr2 JXM region specifically co-immunoprecipitated with IP_3_R1 (Fig. 3*I*). Thus, the Caspr2 JXM domain is both necessary and sufficient to mediate the interaction with IP_3_R1.

### Caspr2 affects ERK signaling and regulates cell morphology through interaction with IP_3_R1

**Figure 4.**
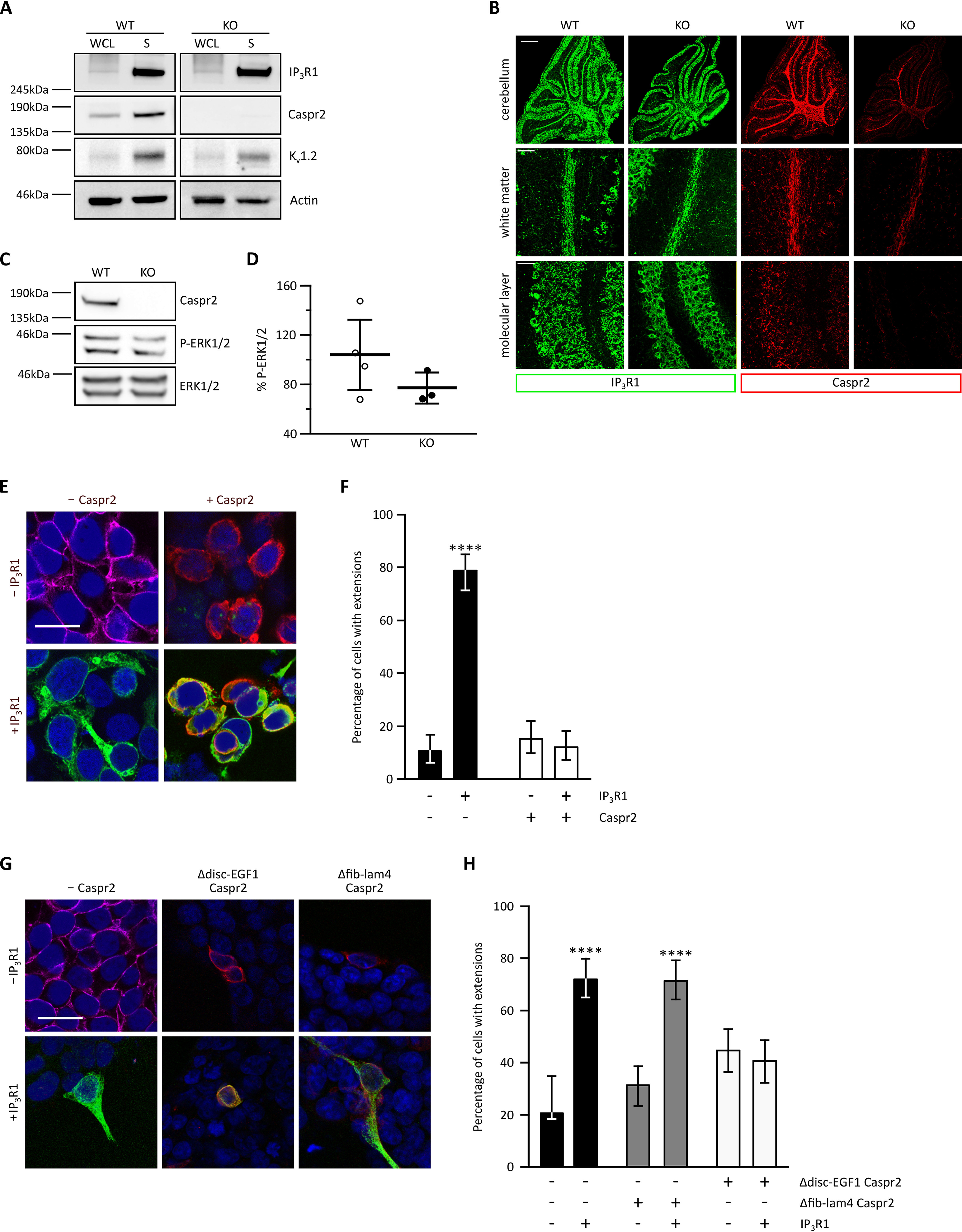
**Caspr2 affects ERK signaling and regulates cell morphology through interaction with IP_3_R1.** The absence of Caspr2 does not perturb the synaptic expression of IP_3_R1. Synaptosomes were isolated from P14 WT and KO mouse cerebellum, lysed, and subjected to immunoblotting for IP_3_R1, Caspr2, the synaptic marker K_v_1.2, and actin. A representative image of five biological replicates is shown. *B*, the absence of Caspr2 does not affect gross IP_3_R1 staining patterns. Shown are representative images of immunostained P14 cerebellar sections from WT and KO animals. Individual sections were co-immunostained with antibodies against IP_3_R1 (*green*) and Caspr2 (*red*). The residual Caspr2 signal observed in the KO sections is likely due to low-level expression of the short Caspr2 isoform (46). *Scale bars*, 500 μm (whole-cerebellum images) and 50 μm (ML and WM images). Images are representative of five independent biological replicates (4 immunostainings/animal). *C*, cerebellar lysates of WT and KO animals were subjected to immunoblotting for Caspr2 and phosphorylated (*P-*) ERK1/2 and ERK1/2. *D*, quantification of P-ERK1/2 levels in KO cerebellum compared with WT littermates. Data were normalized to WT levels and are presented as a scatterplot with mean ± S.D. (*error bars*) superimposed. *n* = 3 (KO) and 5 (WT) animals. *E*, representative images of HEK293FT cells, transfected with the indicated constructs and then immunostained with antibodies against the plasma membrane marker Na^+^K^+^ATPase (*magenta*, *top left*), Caspr2 (*red*), and IP_3_R1 (*green*). Samples were also counterstained with DAPI (*blue*). *Scale bar*, 25 μm. *F*, quantification of the IP_3_R1-induced cellular extension phenotype, which is inhibited by the expression of Caspr2. Cells were categorized as having extensions if some part of the cell was present ≥15 μm away from the edge of the nucleus. *n* = 3 independent replicates for each transfection condition, 50 cells counted per experiment. *Error bars*, 95% confidence intervals. ****, *p* < 0.0001, χ^2^ test with partitioning when comparing the group of conditions marked by *asterisks* with the group of all other conditions. *G*, representative images of HEK293FT cells, transfected with the indicated constructs and then immunostained with antibodies against the plasma membrane marker Na^+^K^+^ATPase (*magenta*, *top left*), Caspr2 (*red*), and IP_3_R1 (*green*). Samples were also counterstained with DAPI (*blue*). *Scale bar*, 25 μm. *H*, quantification of *G*. Caspr2 lacking the IP_3_R1 interaction domain is unable to inhibit the extension phenotype. *n* = 3 independent replicates for each transfection condition, 50 cells counted per experiment. *Error bars*, 95% confidence intervals. ****, *p* < 0.0001, χ^2^ test with partitioning when comparing the group of conditions marked by *asterisks* with the group of all other conditions. *WCL*, whole-cell lysate; *S*, synaptic fraction; *KO*, *Cntnap2* knockout.

We next asked whether Caspr2 is required for the synaptic localization of IP_3_R1 in the cerebellum. We found no reduction of the enrichment of IP_3_R1 in synaptosomes prepared from *Cntnap2* KO mice compared with WT littermates (Fig. 4*A*). Similarly, IP_3_R1 immunoreactivity in the cerebellum was not changed in the absence of Caspr2 (Fig. 4*B*). These findings suggest that Caspr2 is not acting as a scaffold to anchor IP_3_R1 at synapses in the cerebellum. We therefore investigated whether Caspr2 might affect IP_3_R1 function and downstream signaling. We found that the phosphorylation status and thus the activity of the extracellular-regulated kinases 1/2 (ERK1/2) was reduced in *Cntnap2* KO mice compared with WT littermates, consistent with altered intracellular signaling in the absence of Caspr2 (Fig. 4, *C* and *D*). This might be through either Ca^2+^-dependent or -independent modulation of ERK signaling.

Based on our findings that Caspr2 controls Purkinje cell dendritic development (Fig. 2), interacts with IP_3_R1 (Fig. 3), and affects downstream intracellular signaling (Fig. 4, *C* and *D*), we next examined whether the interaction between Caspr2 and IP_3_R1 might mediate cell morphological changes. HEK293FT were used to investigate this hypothesis because the absence of endogenous Caspr2 expression alongside the very low levels of endogenous IP_3_R1 expression in these cells permits a clear-cut rescue experiment. Interestingly, overexpression of IP_3_R1 in HEK293FT cells results in the outgrowth of filopodia-like extensions (Fig. 4, *E* and *F*). Whereas overexpression of Caspr2 alone did not have an effect on cell morphology, the overexpression of Caspr2 together with IP_3_R1 dramatically reduced the number of IP_3_R1-induced extensions (Fig. 4, *E* and *F*). Of note, both Caspr2 and IP_3_R1 appeared to co-occur at the periphery of the cell (Fig. 4*E*), indicating that they communicate at the plasma membrane. Differences in Caspr2 ECD function when it is presented in *cis* and in *trans* may explain why the absence of Caspr2 from the cerebellum potentially decreases calcium levels, whereas the presence of Caspr2 in *cis* inhibits IP_3_R1-induced morphology changes. The Caspr2 Δdisc-EGF1 mutant able to interact with IP_3_R1 (Fig. 3*H*) suppressed the IP_3_R1-induced cellular phenotype (Fig. 4, *G* and *H*). In contrast, the Caspr2 Δfib-lam4 mutant lacking the critical IP_3_R1 interaction domains failed to reduce the number of IP_3_R1-induced extensions (Fig. 4, *G* and *H*). Together, these findings suggest that the specific interaction between Caspr2 and IP_3_R1 acts to inhibit IP_3_R1-induced changes in cell morphology.

## Discussion

In this study, we have uncovered a function for Caspr2 in the developing cerebellum. Loss of Caspr2 impairs Purkinje cell dendritic development and results in motor coordination deficits. We have found that Caspr2 is highly enriched at synaptic specializations in the cerebellum, where it interacts with IP_3_R1. The specific interaction of Caspr2 with IP_3_R1 is mediated by the Caspr2 JXM domain, which plays a critical role in the ability of Caspr2 to inhibit IP_3_R1-mediated changes in cellular morphology. Collectively, our findings define a mechanism by which Caspr2 controls the development and function of the cerebellum.

Members of the neurexin superfamily are thought to function as molecular platforms within the synaptic cleft, where they mediate the formation of protein complexes that control key developmental processes, including synapse organization and neural circuit formation (25, 26). The functions of individual neurexins are context-dependent and largely determined by structural features within their ectodomains and the expression of specific interacting proteins in distinct subsets of synapses. Our study identifies a unique function for Caspr2 in the cerebellum due to the specific interaction of the Caspr2 ECD with IP_3_R1, which is most highly expressed in cerebellar Purkinje cells (19, 27, 28). Notably, the Caspr2-IP_3_R1 interaction does not appear to regulate the synaptic localization of IP_3_R1 in the cerebellum but rather modulates the function of IP_3_R1. IP_3_R1 functions as a calcium channel and is critically important for normal cerebellar function. Mice lacking IP_3_R1 display ataxia (23), impaired cerebellar long-term depression (29), and abnormal Purkinje cell dendritic morphology (30). In light of our findings, at least some of these IP_3_R1 functions are likely to be modulated by Caspr2. In hippocampal neurons, surface expression of Caspr2 is regulated by phosphorylation-dependent endocytosis (31), raising the possibility that Caspr2 function and modulation of IP_3_R1 may be controlled by additional factors such as neuronal activity.

The topology of neurexin proteins has important implications for their function. We here find that Caspr2 is expressed at both pre- and postsynaptic specializations and has functional effects in both in *cis* and in *trans* conformations, raising the possibility that alternate presentations of Caspr2 may have distinct consequences for IP_3_R1 function.

We found that high expression of Caspr2 persists into adulthood, suggesting that Caspr2 might also be important in the mature cerebellum. Interestingly, IP_3_R1 is known to contribute to the maintenance of Purkinje cell spine morphology in adult mice (32). These findings suggest that the Caspr2-IP_3_R1 interaction may regulate critical processes beyond Purkinje cell dendritic development. Together, our findings pave the way for future studies on the downstream effects of the Caspr2-IP_3_R1 interaction in the developing and adult cerebellum and its physiological regulation.

The identification of the synaptic interaction of Caspr2 with IP_3_R1 has important ramifications for calcium signaling in the cerebellum. Although IP_3_R1 is best known as an ER–integral protein, it is also found embedded in the plasma membrane of Purkinje cells and on the postsynaptic density of Purkinje cell dendritic spines (19). Given their large conductances, even a few IP_3_ receptors localized to the plasma membrane can make substantial contributions to Ca^2+^ entry (33). Thus, the synaptic interaction of Caspr2 with IP_3_R1 may provide a key regulatory mechanism for Ca^2+^ entry in Purkinje cells.

Caspr2 is associated with a large number of neurological disorders, including autoantibody-mediated diseases and neurodevelopmental disorders (22). Notably, autoantibodies against Caspr2 have been identified in patients with autoimmune cerebellar ataxia (14–16) but are also associated with a wide range of other PNS and central symptoms, including myotonia and encephalitis (22, 34). These findings raise the interesting possibility that autoantibodies causing different disorders might target different epitopes on Caspr2, thereby influencing the binding of Caspr2 to different critical interaction partners and, by extension, the presenting clinical phenotype.

The cerebellum is emerging as a key brain region in ASD (35, 36), but the underlying molecular, cellular, and circuit mechanisms remain largely to be elucidated. Interestingly, Caspr2 is strongly associated with ASD (7, 8, 10, 37, 38). In the context of our study, these findings raise the intriguing question of whether aberrant function of Caspr2 in the cerebellum might be relevant to ASD. Future studies using conditional *Cntnap2* KO animals will help to determine whether loss of Caspr2 in the cerebellum specifically contributes to ASD behaviors. Interestingly, impaired IP_3_R1-mediated Ca^2+^ signaling might be a shared functional defect in ASD (39). Thus, the interaction of Caspr2 and IP_3_R1 in the cerebellum might provide important clues for the understanding of the molecular mechanisms underlying ASD and related disorders.

## Experimental procedures

### Plasmids

The full-length mouse Caspr2 plasmid was obtained from OriGene (Uniprot ID: E9QNF7, Rockville, MD, USA), and the full-length human Caspr2 plasmid (UniProt ID: Q9UHC6) was a kind gift from Angela Vincent. The Caspr2 ECD construct (residues 28–1262, UniProt ID: E9QNF7) was created by PCR cloning of the respective nucleotide sequence into the pHL-Avitag3 vector (40). The Caspr2 JXM plasmid (residues 592–1257, UniProt ID: Q9UHC6) plasmid (UniProt ID: Q9UHC6) was created by subcloning the relevant base sequence into the 3XFLAG-CMV-10 vector (Sigma). The Caspr2 Δdisc-EGF1 and Δfib-lam4 (Q9UHC6) plasmids have been de-scribed previously (41). The full-length rat SI-SII-SIII-IP_3_R1 (AAA41357.1) construct was kindly provided by Greg Mignery (42). All constructs were verified by Sanger sequencing.

### Animals

Heterozygous B6.129(Cg)-Cntnap2^2m1Pele^/J mice were ob-tained from the Jackson Laboratory and used to establish a colony. Wherever possible, littermates were used when comparing KO and WT mice. WT mice were also obtained from a separate C57BL/6J colony when experiments involved the use of WT animals only. Except where indicated specifically, mice of mixed gender were used. All animal experiments in this work were carried out in accordance with the animals (scientific procedures) act (ASPA) 1986 under Home Office Project licenses 30/3353 and 30/3301.

### Behavioral analysis

Static rod testing was carried out in *Cntnap2* WT, HET, and KO littermates at 5–6 months of age. Mice were placed on the far end of a 60-cm-long rod of 6- and 10-mm diameter, respectively, facing away from the platform. The time taken for the animal to turn around and face the platform was recorded. Two trials were performed per animal per rod diameter and averaged for analysis. A maximum time of 180 s was allowed for completion of the test. Gait analysis was carried out on the same animals using the CatWalk XT walkway (Noldus). Three time points were assessed: 5–6 weeks, 3 months, and 5–6 months. Three uninterrupted runs were recorded per animal, and the average parameters over the three runs recorded per animal were used for statistical analysis.

### Cell culture and transfection

HEK293FT cells (Invitrogen) were grown in DMEM-GlutaMAX (Gibco) supplemented with 10% fetal bovine serum (Gibco) and penicillin/streptomycin (Gibco) and maintained in 5% CO_2_ at 37 °C in humidified conditions. Cells were transfected with Fugene HD (Promega) according to the manufacturer's instructions. For protein production, cells were cultured as described (40).

### Organotypic slice cultures

Organotypic slice cultures were prepared from P9 mouse pups as described (43). Dosing with recombinant Caspr2 ECD (20 µg/ml) or vehicle (10 mm Tris, pH 8, 200 mm NaCl) was carried out after 1 day *in vitro* and then after every change of medium. Slices were cultured for a total of 8 days and then fixed in ice-cold acetone for 5 min, rehydrated in PBS, and then blocked in 10% normal goat serum (Sigma), 0.3% Triton X-100 (Sigma) in PBS before incubation with mouse anti-IP_3_R1 (Santa Cruz Biotechnology, Inc.) antibody overnight at 4 °C, followed by a 2-h incubation with Alexa Fluor–conjugated secondary antibody (Invitrogen) and mounting using Vectashield mounting medium containing DAPI (Vector Laboratories).

### Immunostaining

Dissected cerebella were embedded in optimum cutting temperature compound (Agar Scientific) and frozen on dry ice. 14-µm-thick sagittal sections were cut and stored at −80 °C until required. For immunohistochemistry, sections were left to defrost at room temperature for 10 min before immunostaining as described for the organotypic slice cultures using rabbit anti-Caspr2 (Abcam) and mouse anti-IP_3_R1 (Santa Cruz Biotechnology) primary antibodies. For Fig. 1, stained sections were visualized using a Zeiss Axiovision fluorescence microscope and the AxioVision 4.3 software package. For all other figures, an Olympus FluoView3000 laser-scanning confocal microscope and the FV3000 software module were used.

Transfected HEK293FT cells were fixed by immersion in ice-cold acetone for 5 min, permeabilized in 0.4% (v/v) Triton X-100/1× PBS for 20 min, and blocked using 10% skimmed milk powder, 1% normal goat serum, 1× TBST (150 mm NaCl, 10 mm Tris, pH 8, 0.02% Tween 20 (Bio-Rad)) and immunostained overnight at 4 °C with rabbit anti-Caspr2 (Sigma), mouse anti-IP_3_R1 (Santa Cruz Biotechnology), and mouse anti-Na^+^K^+^ATPase (Abcam) antibodies followed by a 2-h incubation with Alexa Fluor–conjugated secondary antibody (Invitrogen) and mounting using Vectashield mounting medium containing DAPI (Vector Laboratories). Stained cells were visualized using a TCS SP5II confocal microscope (Leica Microsystems CMS GmbH) and Leica application suite software.

### Purification of recombinant Caspr2 ECD

Recombinant Caspr2 ECD was biotinylated *in vivo* and then secreted by transiently transfected HEK293T cells as described (40). For large-scale preparations, conditioned medium was collected 4 days after transfection, centrifuged, filtered, and then diafiltrated into 20 mm Tris, pH 7.6, 1× PBS, and 150 mm NaCl. Recombinant protein was then purified by nickel affinity chromatography, using an HP HisTrap 5-ml column (GE Healthcare), followed by size-exclusion chromatography using a HiLoad 16/600 Superdex 200 pg column (GE Healthcare). The gel filtration buffer was 10 mm Tris, pH 8, 200 mm NaCl.

For small-scale preparations, conditioned medium was harvested 2 days post-transfection, clarified by centrifugation, and then buffered using Tris, pH 8, up to a final Tris concentration of 10 mm. Nickel Excel beads (GE Healthcare) were used to purify recombinant Caspr2 ECD. Protein was eluted from the nickel beads using 500 mm imidazole, 10 mm Tris, pH 8, 150 mm NaCl.

### Biochemical assays

Cerebellar synaptosomes were generated using Syn-PER synaptic protein extraction reagent (Thermo Fisher Scientific), as per the manufacturer's instructions. These were further fractionated to produce pre- and postsynaptic specialization fractions as described (44).

Cells, tissue, and synaptosomes were lysed in hypo-osmotic shock buffer (150 mm NaCl, 10 mm Tris, pH 8, 1× Complete protease inhibitors, and 1× PhosSTOP (both from Roche Applied Science)), homogenized by sonication (HEK293FT) or using a Dounce homogenizer (cerebellum), and centrifuged at 16,000 × *g* for 20 min at 4 °C. The resulting supernatant (whole-cell lysate) was transferred to a new tube and boiled with SDS sample buffer.

For co-immunoprecipitation experiments, lysates were incubated with 1 µg of primary antibody (rabbit anti-Caspr2 antibody (Sigma), goat anti-IP_3_R1 antibody (Santa Cruz Biotechnology)) for 2 h at 4 °C with gentle mixing, followed by the addition of 30 µl of equilibrated IgG-coated Dynabeads (Thermo Fisher) for another 1 h. The beads were then washed three times in ice-cold hypo-osmotic shock buffer and boiled in SDS-PAGE sample buffer.

Standard SDS-PAGE and immunoblotting were carried out. Protein concentrations were determined using the Bradford method. Equal amounts of protein were loaded per lane, up to a maximum of 30 µg. Antibodies used for immunoblotting were as follows: mouse anti-IP_3_R1 (Neuromab), rabbit anti-Caspr2 (Sigma), mouse anti-actin (Abcam), anti-mouse GRID2 (Santa Cruz Biotechnology), anti-rabbit VGluT1 (Synaptic Systems), anti-mouse PSD95 (Synaptic Systems), anti-rabbit syntaxin (Synaptic Systems), anti-rabbit anti-ERK (Cell Signaling), rabbit anti-pERK (Cell Signaling), anti-FLAG (Sigma), and anti-Kv1.2 (Neuromab). Horseradish peroxidase–linked secondary antibodies were from GE Healthcare. Background signal was subtracted from some immunoblots using ImageJ (National Institutes of Health). Densitometry on immunoblots was also carried out using ImageJ.

### Identification of synaptic Caspr2-interacting proteins via LC–MS-MS

Cerebellar synaptosomes, prepared from P14, WT mice, were lysed in hypo-osmotic shock buffer. Synaptosome lysates were then incubated with magnetic MyOne streptavidin-coated dynabeads (Thermo Fisher Scientific) coated with recombinant Caspr2 ECD. Following four washes with hypo-osmotic shock buffer, synaptic proteins bound to Caspr2 ECD were separated from unbound proteins using a magnet and then eluted by boiling in reducing NuPAGE sample buffer (Thermo Fisher). Bound bait protein was then eluted by the addition of 95% formamide, 80 mm NaOAc, followed by further boiling. Protein samples were resolved using SDS-PAGE and then stained using a Coomassie dye. Each gel lane was cut into five sections and processed by in-gel digestion comprising reduction, alkylation, and digestion with trypsin (sequencing grade; Promega) as described previously (45). Trypsin cleaves the C terminus of Arg and Lys residues, except when those residues are followed by Pro. Dried in-gel digests were resuspended in 2% acetonitrile, 0.1% formic acid prior to HPLC injection. An Ultimate 3000 Nano-HPLC system (Dionex, Sunnyvale, CA, USA) was used for the liquid chromatography separation. In-gel digested material was loaded onto a trapping column (Dionex, 300-μm inner diameter, 0.1 cm) at a flow rate of 20 μl/min to facilitate initial concentration. Separation was achieved using a C18 Pepmap column (Dionex, 75-μm inner diameter, 15 cm) and a flow rate of 250 nl/min. A two-solvent gradient buffer was used for elution, where solvent A was 98% H_2_O, 2% acetonitrile, 0.1% formic acid and solvent B was 80% acetonitrile, 20% water, 0.1% formic acid. The proportion of solvent B present in the buffer was gradually increased from 2 to 50% over the course of 30 min followed by column wash (90% B) and re-equilibration. The HPLC system was interfaced directly with a 3D high-capacity ion trap mass spectrometer (amaZon; Bruker Daltonics). Ion trap target mass was set at 850 *m/z*, compound stability at 100%, and smart ICC at 250,000. HyStar software (version 3.2) was used to initiate MS/MS analysis upon detection of a contact closure signal. Helium gas, with a 30–200% collision energy sweep of amplitude 1.0, was used to produce collision-induced dissociation. Five or fewer precursor ions were selected per cycle with active exclusion (0.5 min).

### Data processing and statistics

Mouse behavior (in version 3.2.4) and cell morphology data (in version 3.2.0), were all analyzed in R (R Core Team). Clopper–Pearson 95% confidence intervals were calculated using the R binom package. Comparative statistics were computed using Graph Pad Prism (GraphPad Software). Where parametric tests were used, normality was first confirmed via the Shapiro–Wilk test. To assess the potential co-occurrence of Caspr2 and IP_3_R1 in the cerebellum, Mander's coefficients of co-occurrence (24) were calculated for individual images of co-stained cerebella sections using the ImageJ (National Institutes of Health) coloc2 plugin. The region selected for analysis was restricted to the deep portion of the ML, where a fibrous Caspr2 staining pattern was clearly visible. The Manders coefficients reported for each of the individual images were then averaged. Costes significance tests were also performed simultaneously, alongside the coefficient calculations, using 100 randomizations. A Costes *p* value of 1 was reported for all images examined.

DataAnalysis 4.0 (Bruker Daltonics) was used to produce Mascot-compatible (.mgf) files from raw LC–MS/MS data. Mascot (Matrix Science, algorithm version 2.5.1) was used to perform database searches of the Uniprot *Mus musculus* database (version 2020.04.22, which contains 63,656 entries). Variables were set to the following: 2+, 3+, and 4+ ions; peptide mass tolerance, 0.3 Da; ^13^C = 2; fragment mass tolerance 0.6 Da; number of missed cleavages 2; instrument type ESI-TRAP; fixed modifications, carbamidomethylation (Cys); variable modifications, oxidation (Met). The false discovery rate was set at 5% using a decoy database search, resulting in peptide spectrum match significance levels of *p* < 0.026 and *p* < 0.046 for the Caspr2 ECD and mock IPs, respectively. A minimum expectation score filter of 0.05 was also applied.

## Data availability

All data are contained within the article. The MS proteomics data have been deposited to the ProteomeXchange Consortium via the PRIDE partner repository with the data set identifier PXD018972.

## Supplementary Material

Supporting Information
