# Supplementary material for: Caspr2 interacts with type 1 inositol 1,4,5-trisphosphate receptor in the developing cerebellum and regulates Purkinje cell morphology
Source: J Biol Chem. 2020 Jul 16;295(36):12716–26. doi: 10.1074/jbc.RA120.012655 (PMC7476715; doi:10.1074/jbc.RA120.012655)
Supplement: Supporting Information [file supp_295_36_12716__index.html]

Caspr2 interacts with type 1 inositol 1,4,5-trisphosphate receptor in the developing cerebellum and regulates Purkinje cell morphology — Caspr2 regulates cerebellar development and function — Supporting Information 

# Caspr2 interacts with type 1 inositol 1,4,5-trisphosphate receptor in the developing cerebellum and regulates Purkinje cell morphology

## Supporting Information

- Supporting Information (to be published online) - Supporting Information
- Supporting Information (to be published online) - Supplemental Table S1
