# Supplementary material for: Caspr2 interacts with type 1 inositol 1,4,5-trisphosphate receptor in the developing cerebellum and regulates Purkinje cell morphology
Source: J Biol Chem. 2020 Jul 16;295(36):12716–26. doi: 10.1074/jbc.RA120.012655 (PMC7476715; doi:10.1074/jbc.RA120.012655)
Supplement: Supporting Information [file supp_RA120.012655_158236_2_supp_558110_qdkj1p.pdf]

**SUPPORTING INFORMATION**

# Caspr2 interacts with type 1 inositol 1,4,5-trisphosphate receptor in the developing cerebellum and regulates Purkinje cell morphology

**Liam Argent[1], Friederike Winter[1], Imogen Prickett[1], Maria Carrasquero-Ordaz[2], Abby L. Olsen[3], Holger Kramer[1], Eric Lancaster[3], and Esther B. E. Becker[1]***

From the [1]Department of Physiology, Anatomy and Genetics, University of Oxford, Oxford, OX1 3PT, United Kingdom; [2]Department of Biochemistry, University of Oxford, Oxford, OX1 3QU, United Kingdom; [3]Department of Neurology, University of Pennsylvania, Philadelphia, PA 19104, United States of America

**Running title: Caspr2 regulates cerebellar development and function**

* To whom correspondence should be addressed: Esther B. E. Becker: Department of Physiology, Anatomy and Genetics, University of Oxford, Oxford, OX1 3PT, United Kingdom; esther.becker@dpag.ox.ac.uk; Tel. +44 1865 285866.

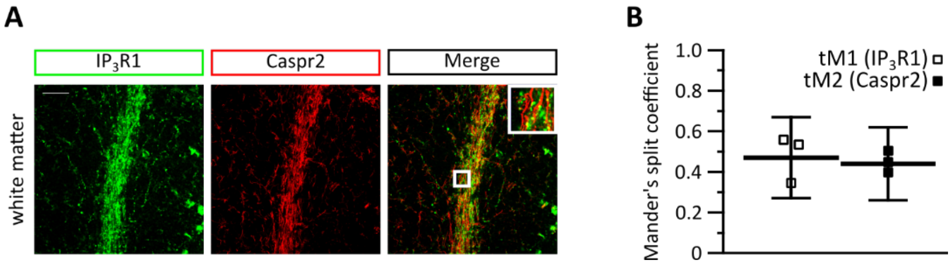


**Figure S1. Caspr2 and IP$_3$R1 do not co-occur in the white matter of the cerebellum.**
(A) Representative images of immunostained P14 cerebellar sections. Individual sections were double-immunostained with antibodies against Caspr2 (red) and IP$_3$R1 (green). Scale bar = 50µm, zoomed inset is 4X magnified. (B) Average white matter Mander's split coefficients ± SD: tM1 (IP$_3$R1) = 0.470 ± 0.200, tM2 (Caspr2) = 0.441 ± 0.183. n = 3 (WT) animals, 4 unique images per animal.

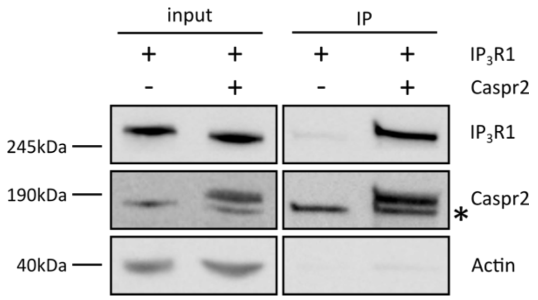


**Figure S2. The Caspr2-$IP_3R1$ interaction is not affected by $Ca^{2+}$ levels.**
Lysates of HEK293FT cells transfected with $IP_3R1$ alone or together with Caspr2 were subjected to co-immunoprecipitation in the presence of 5mM EGTA, which was added to chelate $Ca^{2+}$ ions, with an anti-Caspr2 antibody followed by immunoblotting for Caspr2, $IP_3R1$ and Actin. Nonspecific immunoreactivity is indicated by (*). Images are representative of two independent biological replicates.

**Table S1. LC-MS/MS analysis of pull-down experiment.**
Protein hits reported when MASCOT was used to search the Uniprot "*Mus musculus*" database (v2020.04.22), with the false discovery rate set at 5%. A minimum expectation score filter of 0.05 was also applied. Hits with no significant distinct sequences were excluded.
